# FoxO1 regulates allergic asthmatic inflammation through regulating polarization of the macrophage inflammatory phenotype

**DOI:** 10.18632/oncotarget.8162

**Published:** 2016-03-17

**Authors:** Sangwoon Chung, Tae Jin Lee, Brenda F. Reader, Ji Young Kim, Yong Gyu Lee, Gye Young Park, Manjula Karpurapu, Megan N. Ballinger, Feng Qian, Luiza Rusu, Hae Young Chung, Terry G. Unterman, Carlo M. Croce, John W. Christman

**Affiliations:** ^1^ Pulmonary, Allergy, Critical Care and Sleep Medicine, Ohio State University Wexner Medical Center, Davis Heart and Lung Research Institute, Columbus, Ohio, USA; ^2^ Department of Molecular Virology, Immunology and Medical Genetics and Comprehensive Cancer Center, Ohio State University, Columbus, Ohio, USA; ^3^ Department of Medicine, Section of Pulmonary, Critical Care, and Sleep Medicine, University of Illinois at Chicago, Chicago, Illinois, USA; ^4^ Molecular Inflammation Research Center for Aging Intervention (MRCA), College of Pharmacy, Pusan National University, Gumjung-gu, Busan, Korea; ^5^ Section of Endocrinology, Diabetes and Metabolism, Department of Medicine, University of Illinois at Chicago College of Medicine, Chicago, Illinois, USA

**Keywords:** FoxO1, asthma, eosinophilic lung inflammation, M2 macrophage phenotype, Immunology and Microbiology Section, Immune response, Immunity

## Abstract

Inflammatory monocyte and tissue macrophages influence the initiation, progression, and resolution of type 2 immune responses, and alveolar macrophages are the most prevalent immune-effector cells in the lung. While we were characterizing the M1- or M2-like macrophages in type 2 allergic inflammation, we discovered that FoxO1 is highly expressed in alternatively activated macrophages. Although several studies have been focused on the fundamental role of FoxOs in hematopoietic and immune cells, the exact role that FoxO1 plays in allergic asthmatic inflammation in activated macrophages has not been investigated. Growing evidences indicate that FoxO1 acts as an upstream regulator of IRF4 and could have a role in a specific inflammatory phenotype of macrophages. Therefore, we hypothesized that IRF4 expression regulated by FoxO1 in alveolar macrophages is required for established type 2 immune mediates allergic lung inflammation. Our data indicate that targeted deletion of FoxO1 using FoxO1-selective inhibitor AS1842856 and genetic ablation of FoxO1 in macrophages significantly decreases IRF4 and various M2 macrophage-associated genes, suggesting a mechanism that involves FoxO1-IRF4 signaling in alveolar macrophages that works to polarize macrophages toward established type 2 immune responses. In response to the challenge of DRA (dust mite, ragweed, and *Aspergillus*) allergens, macrophage specific FoxO1 overexpression is associated with an accentuation of asthmatic lung inflammation, whereas pharmacologic inhibition of FoxO1 by AS1842856 attenuates the development of asthmatic lung inflammation. Thus, our study identifies a role for FoxO1-IRF4 signaling in the development of alternatively activated alveolar macrophages that contribute to type 2 allergic airway inflammation.

## INTRODUCTION

Asthma affects more than 26 million people in the United States [1] and is the most common chronic disease of children and young adults^1^. Fundamentally, asthma is an immune disorder of the conducting airways and alveolar spaces that is triggered by exposure to a sensitizing allergen and results in derangement of pulmonary function leading to episodic and ultimately chronic pulmonary disability [2, 3]. The pathology of asthma is generally considered to be a type 2 immune reaction that is characterized by eosinophilic airway inflammation that is associated with reversible airflow obstruction and bronchial hyper-reactivity [4, 5]. The heterogeneous nature of the various asthmatic phenotypes suggests the involvement of complex cytokine networks, which exhibit both agonistic and antagonistic influence on the type 2 immune response [6]. The type 2 immune response induces an inflammatory response characterized by eosinophils, mast cells, basophils, type 2 innate lymphoid cells, and T helper 2 (T_H_2) cells, and IL-4- and/or IL-13-conditioned macrophages [7]. Growing evidence indicates that inflammatory monocyte and tissue macrophages influence the initiation, progression, and resolution of type 2 immune responses [6, 8-10]. Approaches that interrupt the recruitment [9, 11, 12] and/or maintenance [10, 13] of monocyte/macrophages populations in diseased tissues could be developed as novel therapeutic strategies for asthma and a wider range of type 2-driven diseases.

Alveolar macrophages are the most abundant leukocytes found in alveoli, distal airspace, and conducting airway [14], suggesting that they have an important role in maintaining airway immune homeostasis [15]. Macrophages have recently been characterized based on their role in various disease processes and their cytokine production profile as being pro-inflammatory M1 or counter-inflammatory/pro-asthmatic M2 subsets. Macrophages generated in the presence of IFNγ or lipopolysaccharide (LPS) have been termed M1 [16], while M2 macrophages are involved in T_H_2 cytokines IL-4- and/or IL-13 [17]. These subsets show distinct gene expression profiles and functional capabilities [7, 18]. Indeed, our work and that of other groups have established a potential link between lung macrophages and eosinophilic inflammation [8, 9, 19], and airway remodeling [19, 20] in asthma. Thus, T_H_2 cytokines IL-4- and/or IL-13-activated macrophages have important homeostatic functions but also are intimately involved in the control of type 2-driven inflammation and immunity [7, 14]. Although marked progress has been made in the past few years regarding the immunoregulatory function of alveolar macrophages in asthma, the mechanisms that regulate the acquisition of particular functional states in terms of gene expression are just emerging. For example, both M1 and M2 functional characteristics are dependent on the “master myeloid” transcription factor, PU.1 [21, 22], whereas the transcription factors STAT6 and Interferon Regulatory Factor (IRF)4 promote M2 polarization [23, 24], and Krüppel-like factor 4 (KLF4) expression is robustly induced in M2 macrophages [25]. While transcriptional control of macrophages polarization is the subject of intense investigation, the determinants of their speciation and mechanism of action are incompletely understood but probably involve combinatorial interactions among several classes of transcriptional regulatory proteins and epigenetic mechanism.

The forkhead box proteins, O (FoxO) family of transcription factors play key roles in a number of cellular processes, including cell growth, metabolism, survival, and inflammation [26-29]. FoxOs, when present in the nucleus and interact directly with DNA binding site that contain FoxO consensus motif, can act as transcriptional activators and/or repressors [27, 30]. In mammals, the FoxO subclass consists of four members including FoxO1, FoxO3, FoxO4, and FoxO6 [26]. Although several studies have been focused on the fundamental role of FoxO3 in hematopoietic and immune cells, a mechanistic role for FoxO1 in allergic asthmatic inflammation in activated macrophages has not been reported. Our published work indicates that FoxO1 has transcriptional influence on the polarization of macrophages toward an M2-like phenotype [29]. While we were characterizing the M1- or M2-like macrophages, we discovered that FoxO1 is highly expressed in M2-like macrophages, suggesting that FoxO1 might have greater impact on the function of M-CSF- rather than GM-CSF-dependent macrophages [29]. Furthermore, FoxO1 has been regarded as an upstream regulator of IRF4, which is well known to influence M2 polarization [31, 32] and could therefore, we reasoned that FoxO1 could have a pivotal role in the molecular pathogenesis of allergic asthmatic inflammation although this has not been reported by other groups. In order to address whether FoxO1 regulated a pro-asthmatic macrophage phenotype and the type 2 immune response, we utilized genetic and pharmacological approaches, including development of macrophage-specific FoxO1-deficient and -overexpressing mice that were test in the DRA model which exhibits impressive asthmatic airway changes [8, 33] including peribronchial and alveolar eosinophilia [8, 34]. Here we provide striking evidence that FoxO1 is centrally involved in the IRF4 expression and the development of allergic asthmatic lung inflammation in the triple allergens (dust mite, ragweed and aspergillus, DRA)-induced mouse model of allergic asthmatic inflammation. Our data indicate that targeted deletion of FoxO1 using either a FoxO1-selective inhibitor AS1842856 or genetic ablation of FoxO1 in macrophages markedly attenuates IRF4 and M2 macrophage-associated gene expression. Collectively, these findings indicate that FoxO1-IRF4 signaling in alveolar macrophages contributes to the type 2 immune response that results in allergic asthmatic inflammation and that pharmacologic inhibition of FoxO1 could be developed as a novel therapeutic approach for treating refractory asthma through regulation of T_H_2-mediated airway inflammation.

## RESULTS

### FoxO1 protein expression is increased in alternately activated alveolar macrophages

**Figure 1:**
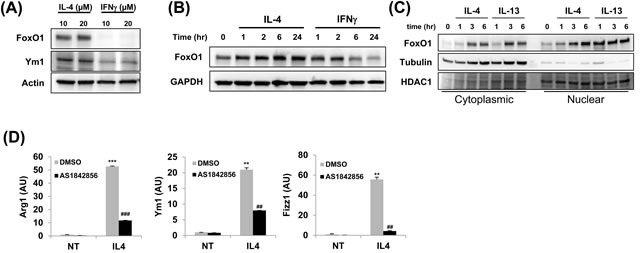
FoxO1 is increased in alternately activated alveolar macrophages **A.** FoxO1 expression in different subsets of MH-S alveolar macrophages. MH-S cells were stimulated with IL-4 or IFN-γ for 24 h. FoxO1 is virtually eliminated in cells by IFN-γ. **B.** Immunoblot of FoxO1 in control or IL-4 or IFN-γ-treated macrophages for 0-24 h. **C.** Cytoplasmic and nuclear extracts were prepared from IL-4 and IL-13-stimulated MH-S alveolar macrophages, and FoxO1 localization was measured by Western blot analysis. **D.** qPCR analysis of Arg1, Ym1, Fizz1 mRNA expression by MH-S pretreated with FoxO1 inhibitor AS1842856 (1μM) and stimulated with for 24 h with IL-4 (10 ng/ml). Data are representative of at least three independent experiments (A-D). **p < 0.01, ***p < 0.001 *vs*. nontreated, ##*p* < 0.01, ###*p* < 0.001 *vs*. IL-4 with noninhibitor (student's *t*-test).

We have recently reported that FoxO1 contributes polarization of macrophages toward the M2-like phenotype and is highly expressed in M-CSF-derived (M2-like) macrophage subsets [29]. Furthermore, other groups have shown that IFNγ antagonizes overall FoxO1 dependent transcriptional program through posttranslational modifications by targeting its acetylation levels [35]. To determine whether FoxO1 regulation occurs in M1 and M2 subsets differentially, we stimulated MH-S alveolar macrophages with IL-4 and/or IFNγ and analyzed them for FoxO1. We noted that FoxO1 protein expression is differentially expressed in IFNγ-treated M1 macrophages in compare to IL-4-treated M2 macrophages. We observed a significant decrease in FoxO1 in cells treated with IFNγ, whereas, IL-4 treatment resulted in higher levels of FoxO1 by prevented degradation of FoxO1 (Figure 1A). At each time point examined, protein level of FoxO1 was attenuated in the presence of IFNγ. In contrast, FoxO1 expression was stimulated by IL-4 (Figure 1B). Moreover, we determined an increase in nuclear compartmentation of FoxO1 in alternatively compared to classically activated MH-S alveolar macrophages (Figure 1C). To test whether FoxO1 acting in macrophages has a potential role in M2 subset, we treated bone marrow derived-macrophages (BMDMs) with FoxO1-selective inhibitor AS1842856 [27, 36]. In accordance with decreased FoxO1 activity, M2-related Arg1, Ym1, Fizz1 genes were markedly decreased compared to the vehicle control group of BMDMs (Figure 1D). Taken all together, these data support a conclusion that FoxO1 has an important role in regulating the generation of alternatively activated M2 alveolar macrophages, but not M1 macrophages.

### FoxO1 regulates IRF4 gene expression in IL-4-stimulated macrophages

**Figure 2:**
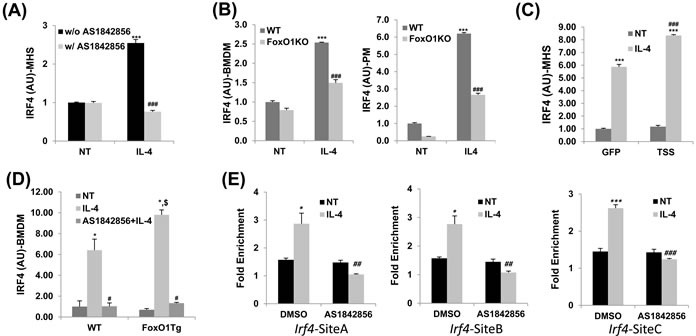
IRF4 expression regulated by FoxO1 in IL-4 stimulated macrophages **A.** Pretreatment with AS1842856, a selective FoxO1 inhibitor blunted the upregulation of IRF4 transcript in IL-4-activated MH-S alveolar macrophages. **B.** qPCR analysis of IRF4 in bone marrow derive macrophages (BMDM) and peritoneal macropahges (PM) from WT and LysMFoxO1KO mice. **C.** qPCR analysis of IRF4 by IL-4 stimulated M2 macrophages infected with an adenovirus expressing either GFP or FoxO1 TSS (where TSS is a mutant containing the mutations T24A, S256A and S319A). **D.** qPCR analysis of IRF4 in IL-4 stimulated BMDM of LysMFoxO1Tg mice. **E.** ChIP assay of FoxO1 binding to Irf4 promoter constructs in MH-S alveolar macrophages in the presence or absence of AS1842856. All results are normalized to IgG. Data are representative of at least two independent experiments (A-E). *p < 0.05, **p < 0.01, ***p < 0.001 *versus* nontreated, #*p* < 0.05, ##*p* < 0.01, ###*p* < 0.001 *vs*. IL-4 with noninhibitor, $*p* < 0.05 *vs*. IL-4 treated WT (student's *t*-test).

IL-4, a classic inducer of the M2 phenotype, increases gene expression of IRF4 that is a critical transcriptional regulator of M2 polarization in macrophages [23]. IRF4 has been studied in the context of immune regulation and has been shown to be involved in lymphoid, myeloid, and dendritic cell development [37-39]. There is already growing evidences indicate that FoxO1 acts as an upstream regulator of IRF4 and therefore could have an influence on develop of the M2 macrophage inflammatory phenotype [31, 32]. Thus, we next investigated a potential link between FoxO1 and the IRF4-regulated M2 polarization program in macrophages. Treatment of IL-4-stimulated alveolar macrophages with AS1842856 (1μM), a highly specific pharmacologic FoxO1 inhibitor, markedly decreased mRNA expression of IRF4 (Figure 2A).

To assess this in the context of IL-4 stimulation, we performed the following loss- and gain-of-function experiments. We generated mice with deletion and/or overexpression of FoxO1 in macrophages by breeding floxed FoxO1 [29] or FoxO1CA [40] mice with LysMCre-expressing mice. IRF4 mRNA levels in IL-4-stimulated macrophages were blunted in myeloid-specific FoxO1 deficient mice (FoxO1 KO; Figure 2B). We next performed a reciprocal ‘gain of function’ experiment, where we overexpressed a constitutive, active form of FoxO1 with an adenoviral expression construct [29] in MH-S cells. In this experiment, MH-S were transfected with an adenovirus expressing GFP or FoxO1 TSS and stimulated with IL-4 (10 ng/ml) for 24 h. Notably, FoxO1 overexpressed MH-S cells (TSS, Figure 2C) and FoxO1 transgenic BMDMs (FoxO1Tg, Figure 2D) both displayed augmented IRF4 expression compared to WT cells. Pretreatment with AS1842856 also blunted IL-4 induced IRF4 expression, reversing the difference between WT and FoxO1Tg macrophages (Figure 2D). These changes are important since IRF4 is a responsible for controlling M2 phenotype [23], raising the possibility that FoxO1 could also impact its overall ability to control T_H_2 immune responses.

Our data indicating that FoxO1 was located in the nucleus of M2 macrophages and regulated the transcriptional M2 program led us to hypothesize that FoxO1 might act like a transcription factor in alternatively activated macrophages. By using a conventional chromatin immune- precipitation (ChIP) assay, we observed binding of FoxO1 in the *Irf4* promoter and identified putative FoxO1 binding sites in IL-4-stimulated MH-S alveolar macrophages but not in FoxO1 inhibitor-treated cells (Figure 2E), which demonstrates that FoxO1 could directly induce the transactivation of the *Irf4* gene. These results are strongly supportive of our hypothesis: that IRF4 expression, regulated by FoxO1 in macrophages, contributes to the type 2 allergic asthmatic response.

### Asthmatic lung inflammation is accentuated in mice that harbor a macrophage specific-overexpression of FoxO1

**Figure 3:**
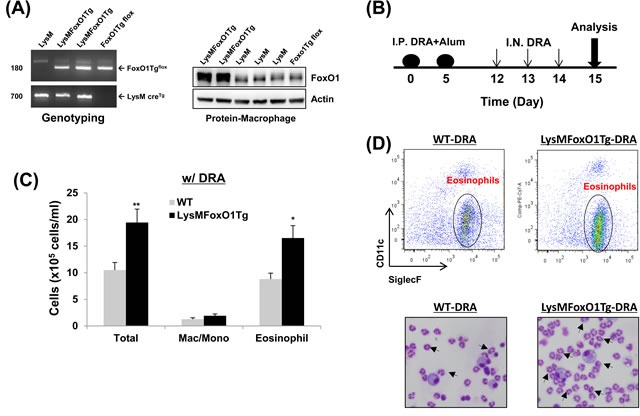
Mice with macrophage specific-overexpression of FoxO1 have enhanced DRA-induced asthmatic response **A.** FoxO1 PCR yields a 180-bp for the FoxO1Tg LoxP allele. A 700-bp for the LysM cre recombinase allele (bottom). Overexpression of FoxO1 protein in peritoneal macrophages from LysM, LysMFoxO1Tg, and FoxO1Tg flox mice. **B.** The schematic timeline showed that the mice were sensitized with DRA on day 0 and 5 and challenged with DRA on day 12, 13, and 14. On day 15, following biomarkers were detected for asthmatic inflammation. **C.** Total cells and macrophages/eosinophils influx in BAL fluid were counted based on total amount of BAL cells, analyzed by flow cytometry. **D.** (Upper) Cell staining for markers of eosinophils (SiglecF^+^CD11c^−^) and macrophages (SiglecF^+^CD11c^+^). (Bottom) Eosinophilic inflammation was enhanced in the LysMFoxO1Tg mice (arrowheads indicate eosinophils). Data are representative of at least three independent experiments (A-D). (*N* = 6-8) *p < 0.05 and **p < 0.01 *vs*. WT-DRA.

**Figure 4:**
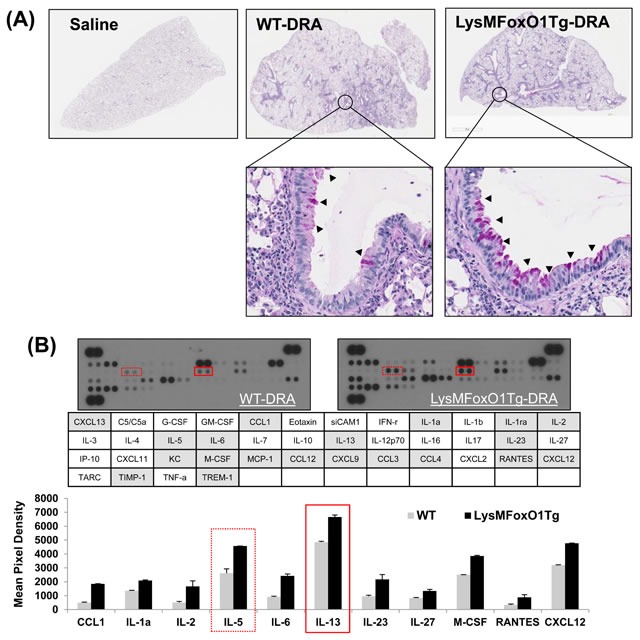
LysMFoxO1Tg mice showed impaired development of DRA-induced allergy airway inflammation **A.** Histopathology was performed based on H&E staining to determine the asthmatic inflammation in Saline- or DRA-treated WT or LysMFoxO1Tg mice. Upper panel shows H&E staining for the entire left lungs. Lower panel shows a zoomed section of the lung as indicated by a square in the upper panel. PAS-stained lung sections from the mice exposed to PBS or DRA. Black arrowheads indicate PAS-positive goblet cells. **B.** Cytokines were detected in BAL fluid from DRA-challenged WT (LysM) and LysMFoxO1Tg mice was quantified and normalized to WT using an antibody array.

Since FoxO1 has not been shown by other groups to be involved in the asthmatic lung inflammation, macrophage-specific FoxO1 transgenic mice (LysMFoxO1Tg, Figure 3A), were subjected to sensitization and challenge with three combined allergens; dust mite, ragweed and aspergillus (DRA) allergens. The DRA model was chosen because it features impressive chronic asthmatic airway changes [33] including peribronchial and alveolar eosinophilia [8, 34]. We subjected LysMFoxO1Tg mice to sensitization and challenge with DRA, according to the protocol shown in Figure 3B [8]. DRA challenge induced an 80% increase in total cell counts (mostly eosinophils, 51.3% to 71.9%, Figure 3C and 3D in these LysMFoxO1Tg mice and this increase was confirmed by histological examination that indicated marked bronchial hyperplasia of periodic acid-Schiff (PAS) positive goblet cells (important mediators of asthmatic lung inflammation), when compared with DRA-challenged WT mice (Figure 4A). No mucin-positive cells were found in either WT or LysMFoxO1Tg mice that were saline exposed. In experiments using macrophage-specific FoxO1 transgenic mice, we observed higher expression of the T_H_2/M2 cytokines in BAL fluid of DRA-challenged compared to their WT counterpart, thus indicating a greater abundance of alternatively activated macrophages in the lungs of these mice (Figure 4B). Shaded cells indicate the cytokines up-regulated compared to DRA-challenged WT counterpart.

### A selective FoxO1 inhibitor, AS1842856, attenuates eosinophilic lung inflammation in sensitized and DRA-challenged WT mice

**Figure 5:**
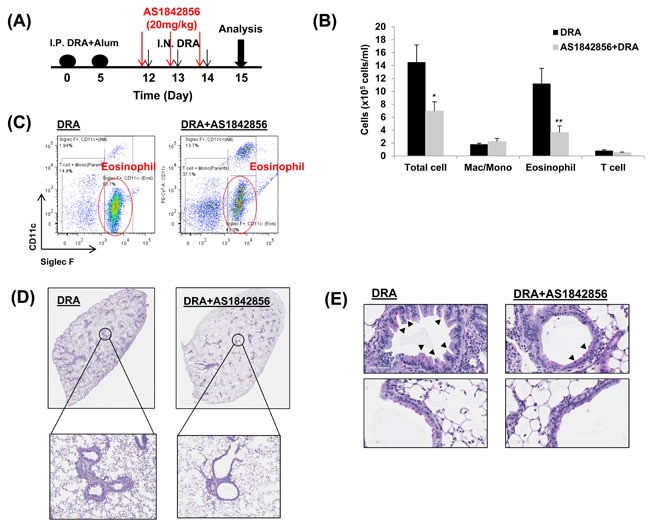
A selective FoxO1 inhibitor, AS1842856, attenuates eosinophilic lung inflammation in sensitized WT mice that is challenged with the DRA allergens **A.** WT mice were subjected to DRA sensitization and challenge as shown in the protocol depicted in Figure 3B. Prior to allergen challenge, mice were treated with vehicle or 20mg/kg of AS1842856, a selective FoxO1 inhibitor. **B.** As shown, eosinophilic inflammation was attenuated in WT mice by pharmacologic inhibition of FoxO1. **C.** Total cells and macrophages/eosinophils influx in BAL fluid were counted based on total amount of BAL cells, analyzed by flow cytometry. **D.** This was confirmed by histology as shown. **E.** PAS-stained lung sections from the mice exposed to PBS or DRA. Black arrowheads indicate PAS-positive goblet cells. Data are representative of at least three independent experiments (A-E). (*N* = 6-8) *p < 0.05 and **p < 0.01 *vs*. DRA.

**Figure 6:**
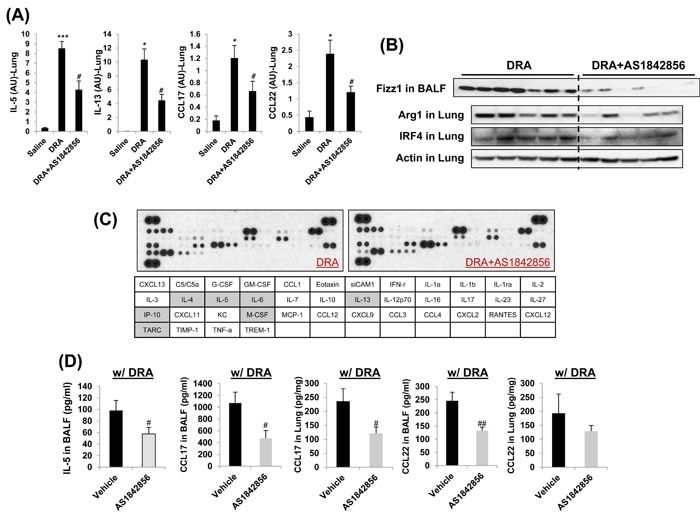
Decreased TH2/M2 immune response was detected in AS1842856 treated-DRA challenge group compared to the only DRA challenge group **A.** Quantitative RT-PCR analysis of gene expression in lung tissues from DRA-challenged mice with or without AS1842856 treatment. **B.** M2-related Fizz1, Arg1, and IRF4 protein expressions are virtually eliminated by pharmacologic inhibition of FoxO1 (AS1842856). **C.** Cytokines were detected in BAL fluid of the AS1842856 treated-DRA challenge group was quantified and normalized to the only DRA challenge group using an antibody array. Highlighted cells indicate the down-regulated cytokines compared to DRA only-challenged counterpart. **D.** CCL17 and CCL22 cytokines were quantified with ELISA in BAL fluid and blood and lung homogenates of AS1842856 pre-treatment group. Data are representative of at least three independent experiments (A-D). *p < 0.05, **p < 0.01, and ***p < 0.001 *vs*. Saline. #*p* < 0.05 and ##*p* < 0.01 *vs*. DRA alone.

Our *in vitro* experiment with MH-S alveolar macrophages treated with AS1842856 resulted in change of IRF4 expression dramatically (Figure 2A). Targeted deletion of FoxO1 using a FoxO1-selective inhibitor, AS1842856, or genetic ablation of FoxO1 in macrophages significantly decreases IRF4 and M2 macrophage-associated genes. To better understand the mechanism whereby FoxO1 inhibition leads to further change *in vivo*, we next treated sensitized WT C57BL/6 mice with AS1842856. DRA-sensitized WT C57BL/6 mice were administered AS1842856 (20 mg/kg) 1h prior to DRA challenge once a day for 3 consecutive days (Figure 5A). Pretreatment with AS1842856 resulted in retention of resident alveolar macrophages and an attenuation of the alveolar eosinophil percentage (80.7% to 43.3%, Figure 5B and 5C) in BAL fluid. This is also confirmed by histological examination with marked bronchial hyperplasia of PAS positive goblet cells (Figure 5D). To investigate possible inflammatory changes in AS1842856-treated mice, we analyzed gene expression in lungs by quantitative real-time PCR. These analyses indicate that pretreatment of DRA-challenged mice with the FoxO1 selective inhibitor results in a significant decrease in the normally augmented expression of IRF4-modulated lung mRNAs like IL-5, IL-13, CCL17/TARC, and CCL22/MDC (Figure 6A). We and others have previously shown that CCL17/TARC and CCL22/MDC have been associated with alternatively activated macrophages [8, 22]. In this case, AS1842856 markedly blunted M2-related Fizz1, Arg1, and IRF4 proteins expression in BAL fluid and whole lung tissue (Figure 6B). From data obtained from a cytokines antibody array, attenuated T_H_2/M2 immune response was detected in BAL fluid of AS1842856 treated-DRA challenge mice compared to the only DRA challenge mice (Figure 6C). Highlighted cells indicate the downregulated T_H_2/M2-related cytokines. Additionally, using ELISAs, there was significance that an increase in macrophages-derived T_H_2 promoting chemokines IL-5, CCL17/TARC, and CCL22/MDC was prevented by AS1842856-treatment (Figure 6C and 6D). These data support an important role for FoxO1 in regulating asthmatic lung inflammation by governing the IRF4 signaling pathway and indicate that FoxO1 inhibition is a potential novel therapeutic approach for treating asthma through regulation of type 2 immune response.

## DISCUSSION

Recently, there is a growing appreciation that “Alternative Activation” of macrophages drives the “M2 macrophage phenotype” that has tissue reparative, cellular proliferative, and angiogenic mechanisms that could be involved in the pathogenesis of asthma [8, 9, 14, 41-43]. Macrophages are not generally considered to be involved in the current asthma paradigm that focuses on T_H_2 cytokine and T-lymphocyte cell mediated events. However, alveolar macrophages are the most abundant resident immune/effector cells in airspace with the potential to have a role in the cellular pathogenesis of asthmatic inflammation and airway remodeling. Yet, so far published studies have yielded ambiguous results regarding the roles of alveolar macrophages in various experimental models of asthma because it appears that macrophages can be both inhibitory and permissive, depending on the exact context and the details of the model system [6]. We believe that this ambiguous role for macrophages in the pathogenesis of asthmatic inflammation is related to the experimental difficulty in distinguishing the role of macrophages and macrophage-derived dendritic cells in allergen sensitization from their role in allergen challenge. Furthermore, even if macrophages have a contradictory role in regulating asthmatic inflammation, scientific information regarding the regulation of these dualistic mechanisms could lead to important therapeutic insight. Our recent studies strongly support the proof of concept that pulmonary macrophages, in response to the dynamic environment of the allergic airway in both human and mice are involved in the pathogenesis of allergic eosinophilic lung inflammation [8, 9, 44]. This is consistent with reports that the removal of alveolar macrophages in mice worsens lung function and type 2 inflammation [45]. Depletion of alveolar macrophages during allergic disease delayed inflammatory resolution and there was a decrease in the production of the immune regulatory cytokines [46]. Moreover, alternatively activated macrophages responded to IL-4 and IL-13, key cytokines in asthma pathology and furthermore, promoted a T_H_2 environment and airway remodeling [43, 47]. Genetic ablation of IL-33 results in less ovalbumin (OVA)-induced airway inflammation associated with less M2 macrophage differentiation, suggesting the importance of M2 macrophages in asthma [48]. Interestingly, alveolar macrophages in OVA-induced airway inflammation, but not T_H_17 cells, are able to produce IL-17 through the up-regulation of chemotactic factors that increase mast cell trafficking while administration of IL-17 neutralizing antibody can suppress inflammatory cell recruitment [49]. Given the relatively long life span of resident pulmonary macrophages and long lasting capacity to produce inflammatory and tissue remodeling mediators, prior studies suggest that macrophages could have a major role in chronic repeated allergic challenge that contributes to severe airway remodeling.

FoxO1 is the most abundant and best-studied member of FoxO isoform. FoxO1 function has been investigated in the tissues and cells of various genetically modified mice of different disease models such as diabetic complications, cardiomyopathy, carcinogenesis, innate immune response, and adaptive immunity [50]. Several lines of studies pointed to a pro-inflammatory role of FoxO1 in inflammatory signaling [51-53]. Most notably, FoxO1 promotes inflammation by increasing expression of several proinflammatory genes, such as IL-1β [52], Tlr4 [51], IL-6, and IL-12 [54]. However, a potential role of FoxO1 in mediating polarization of the M1 and M2 inflammatory phenotype in macrophages has not been fully investigated. We found that FoxO1 is highly expressed in M2-like macrophages, suggesting that FoxO1 might have greater impact on the function of M-CSF- rather than GM-CSF-dependent macrophages [29]. Our data show that FoxO1 is associated with alternatively activated macrophage phenotype and is necessary for regulating M1/M2 polarization. Moreover, it is interesting to note that in previous studies IRF4 expression is induced by FoxO1, allowing for IRF4 to drive transcription of several genes [31, 32, 55]. IRF4 seems to be activated in macrophages as a “brake” on proinflammatory genes expression and “accelerator” on alternatively activated macrophages polarization [55]. Our studies extends these findings by showing that IRF4 expression is regulated by FoxO1 in alveolar macrophages, establishes type 2 immune response in the lung during the allergic asthmatic response. These new findings presented here illustrate that FoxO1 links the alternatively activated macrophages triggered by IL-4, a classic agent of the M2 phenotype, to induced IRF4 activation and this is crucial for M2 macrophages function, in part through of type 2 immune response.

IRF4 has been shown to be involved in the differentiation of most known CD4^+^ T-cells subsets [56]. In allergic airway inflammation, IRF4 is crucial for the regulation of type 2 immune response in mouse model of allergic asthma [57, 58]. Cell specific deletion of IRF4 showed a dramatic defect in T_H_2-type lung inflammation, suggesting this could also be true in alveolar macrophages. In addition, IRF4 has been shown to be a strategic transcription factor in the development and function of various immune cells, including B cells, T cells, and macrophages [59-61]. However, the role of IRF4 in immune responses is not solely confined to T and B lymphocyte cells. IRF4 has been shown to exert a number of selective effects on dendritic cells function [62, 63]. IRF4 is identified as encoding a key transcription factor that controls M2 macrophage polarization [23]. The latter confirmed the prior studies, proposed that the role of IL-4-IRF4 signaling pathway in T helper lymphocytes, suggesting it may also be proper in macrophages. Perhaps most importantly, our data shows an important role for FoxO1 in macrophages in the contribution of allergic disease, and highlight the separate role that FoxO1 could be a major inducer of IRF4 in macrophages, through gain and loss-of-function studies.

There are strong data that show polarization of macrophages to the M2 phenotype is associated with asthmatic inflammation but the role of M2 macrophages in asthmatic inflammation and airway remodeling has not been established. Published studies are ambiguous regarding the roles of macrophages in various experimental models of asthma because it appears that macrophages can be both inhibitory and permissive [45, 64-66]. Whereas dendritic cells are only known to be involved in allergen sensitization, we believe that macrophages have a separate role in allergen sensitization and allergen challenge that has not yet been distinguished. Here we investigated whether FoxO1 participated in macrophage-mediated induction of allergic sensitization to DRA and T_H_2-mediated airway inflammation. LysM-conditional FoxO1-deficient and -transgenic mouse were created to characterize further role of FoxO1 expression by macrophages in allergic lung inflammation. As expected, DRA-challenged macrophage-specific FoxO1 overexpressing mice displayed a phenotype of impaired allergic airway inflammation and accentuated T_H_2-immune responses to DRA that were associated with expression of IRF4, chemokine production, and mucus cell hyperplasia. It is an interesting note that in our preliminary data disrupting FoxO1 in the macrophages of mice with DRA challenge had showed no obvious phenotypic changes (data not shown), and that multiple knock out myeloid FoxOs was necessary to promote oxidative stress and inflammatory responses [67, 68]. While multiple FoxOs proteins may contribute to the regulation of immune response in response to DRA, our results indicate that FoxO1, which is the most abundant isoform in macrophages, is a critical mediator of type 2 immune response in the lung during the allergic asthmatic response. This contrasts sharply with the dramatic effect DRA challenge that results when FoxO1 is overexpressed in macrophages of mice accentuating allergic airway inflammation.

FoxO1 is known to be regulated by phosphorylation and acetylation[51]. Once phosphorylated, FoxO1 is excluded from the nucleus and losing its ability to regulate target genes [27]. Such loss of FoxO1 activity used to explain several molecular mechanisms of these complex disease models [69-71]. Here, we have shown that pharmacologic inhibition of FoxO1 with a highly selective FoxO1 inhibitor, AS1842856 has been shown to markedly attenuate the development of asthmatic lung inflammation. Interestingly, it is known that AS1708727 regulates FoxO1 transcription activity, affecting the FoxO1 transcription-activating domain but not phosphorylation or acetylation [36]. Without manipulation of FoxO1 function, this approach that we chose for our study allow us to focus our intervention strategy for treating asthma through regulation of type 2 immune response *via* FoxO1-IRF4 signaling pathway.

Taken together with our finding showing FoxO1 has a crucial role in up-regulating the alternatively activation of alveolar macrophages, it may well be that an exacerbation type 2 immune allergic airway inflammation in response to allergen challenge. The present finding that FoxO1 as a central effector molecule in the development of allergic inflammation suggests a new therapeutic approach to alleviate the suffering of T_H_2/M2 cell-related allergic diseases.

## MATERIALS AND METHODS

### Materials

Unless otherwise stated, all biochemical reagents used in this study were purchased from Sigma (St. Louis, MO). FoxO1 selective inhibitor AS1842856 was from EMD Millipore (San Diego, CA). Antibody against FoxO1 and IRF4 were purchased from Cell Signaling Technology (Danvers, MA). Antibodies against CD11b and MARCO were purchased from BD Biosciences and R&D system (Minneapolis, MN), respectively. Anti-CD11c, CD3, and SiglecF were purchased from eBiosciences (San Diego, CA). Anti-actin antibody was purchased from Pierce (Rockford, IL).

### Cell cultures

Bone marrow-derived macrophages (BMDMs) from mice were isolated according to published protocols [22, 29] and grown in RPMI1640 supplemented with 10% FBS, 1% penicillin/streptomycin, recombinant mouse macrophage colony-stimulating factor (M-CSF, 10 ng/ml; Peprotech, Rocky Hill, NJ). After 7 days, adherent cells were washed with PBS and replated, then stimulated IL-4 (Peprotech). Mouse alveolar macrophages MH-S cells (ATCC CRL-2019) were cultured in RPMI1640 supplemented with 10% FBS and 1% penicillin/streptomycin.

### Generation of mice with FoxO1-deficient (LysMFoxO1KO) and -overexpressed (LysMFoxO1Tg) myeloid cells

All experiments involving mice were conducted with protocols approved by the Institutional Animal Care and Use Committee (IACUC) of the Ohio State University. To generate myeloid FoxO1^−/−^ mice, FoxO1^fl/fl^ mice [29] were crossed with LysM Cre mice to homozygozity (FoxO1^fl/fl^Cre^Tg^, LysMFoxO1KO). DNA extraction and genotyping were performed as described previously [29]. To avoid the possibility that results could be influenced by Cre recombinases-induced toxicity, FoxO1^wt/wt^Cre^Tg^ mice were used as WT controls.

FoxO1Ca^fl/fl^ (*R26*^*floxneoΔ256FoxO1*^) mice [40] were bred with LysM Cre mice to homozygozity (FoxO1CA^fl/fl^Cre^Tg^, LysMFoxO1Tg). The mice were genotyped by PCR using genomic DNA isolated from tail clippings. The primers for FoxO1Ca^fl/fl^ mice were 5′-ATGGACTACAAAGACGATGAC-3′ (sense) and 5′-GTCGAGTTGGACTGGTTAAAC-3′ (antisense).

### Allergens

Triple allergens (DRA) include extracts of dust mite (*Dermatophagoides farina*), ragweed (*Ambrosia artemisiifolia*), and *Aspergillus fumigates* (Greer Laboratories, Lenoir, NC). Aluminum (Inject Alum; Thermo Scientific) was used for adjuvant. Quantities of allergens for intraperitoneal (100 μl) per mouse were used as follows: *D. farina* (5 μg, 3-35 EU by means of LAL assay), ragweed (50 μg, 5 EU), and *Aspergillus fumigates* (5 μg, 0.1 EU) [22]. Quantities of allergens for intranasal injection (30 μl) were used as follows: *D. farina* (8.3 μg), ragweed (83.4 μg), and *Aspergillus fumigates* (8.3 μg).

### DRA murine asthma model

We used the triple-allergen (DRA)-induced allergic asthma model as previously described [8, 9]. Briefly, mice (8-12 weeks old) were sensitized with the DRA allergen mixture on Days 0 and 5 by intraperitoneal injection with alum (Thermo Fisher Scientific) and then challenged with the DRA mixture at the same concentration used for sensitization on Days 12, 13, and 14 by intranasal delivery. The mice were killed on Day 15, and bronchoalveolar lavage (BAL) fluid and lung tissues were collected for further analysis. AS1842856 (20 mg/kg; EMD millipore) was dissolved in 10% DMSO in PBS and administrated by peritoneal injection 1 hr prior to DRA challenge daily for 3 days. Timelines of the DRA models are shown in Figures 3B and 5A, respectively.

### Lung tissue preparation

Mouse lung tissue was prepared using pressurized low-melting agarose. Briefly, 1.5% wt/vol low-melting-point agarose was boiled at 60°C and then kept at 42°C in water bath. After tracheostomy was performed, the 1.5% melted agarose was infused through the tracheostomy tube from height of 28 cm H_2_O to pressurize equally over lung fields. The tracheostomy tube was tied and lung tissue was put into a formalin container that was refrigerated overnight to facilitate solidification and fixation. Both hematoxylin and eosin (H&E) staining and periodic acid-Schiff (PAS) staining were conducted by the Comparative Pathology and Mouse Phenotyping Shared Resource at the Ohio State University. Slides were scanned using the Aperio ScanScope XT eSlide capture device (Aperio, Vista, CA), and analyzed by Aperio ImageScope digital analysis software (v9.1).

### BAL differential cell count

BAL fluid was collected by lavaging the lung with 800 μl of PBS twice *via* a tracheal catheter and analyzed for total cell counts by countess automated cell counter (Life Technologies). BAL fluid on cytospin slides was stained with HEMA 3 (Thermo Scientific) for differential cell counts. The number of macrophages and eosinophils was quantitated and compared for statistical significance.

### Flow cytometry

Cells collected from BAL fluid were incubated with Fc blocking anti-mouse CD16/32 antibody (BD Bioscience) followed by PE-conjugated anti-SiglecF, FITC-conjugated anti-CD3, PE-Cy7-conjugated CD11c, and APC-conjugated anti-CD11b antibodies. Cells were analyzed on a BD LSR II (BD bioscience) where gating was based on respective unstained cell population and isotype matching control antibodies. The data were analyzed with FlowJo software (TreeStar).

### Measurement of cytokines

The Proteome Profiler™ mouse cytokine array panel A (R&D systems) was used to detect cytokine expression profile in mouse BALF. Cytokine secretion in culture supernatants was analyzed by ELISA specific for mouse IL-5, IL-13, CCL17, CCL22 (R&D systems) following the protocols supplied by the manufacture.

### Western blot analysis

Cells were lysed in RIPA lysis buffer (Millipore, Temecula, CA) with 1 × protease inhibitor cocktail (Pierce). Nuclear-cytoplasmic fractionation was conducted using the NE-PER™ Nuclear and Cytoplasmic Extraction Reagents kit (Thermo Fisher Scientific) according to the manufacturer's protocol. Cell lysates containing equal amount of protein were electrophoresed and immunoblotted using appropriate antibodies as described [21].

### RNA extraction and quantitative real-time RT-PCR

RNA was extracted from cells or lung tissues homogenates by using a miRNeasy Mini kit (QIAGEN) according to the manufacture's instruction. cDNA synthesis with RevertAid First Strand cDNA Synthesis Kit (Thermo) and gene expression was measured by the change-in-threshold (ΔCt) method based on quantitative real-time PCR in an Roche LightCycler 480 (Roche), normalizing to GAPDH expression as an endogenous control.

### Chromatin immunoprecipitation

Chromatin immunoprecipitation assays were performed with SimpleChIP enzymatic ChIP kit (Cell Signaling) with anti-FoxO1 (SantaCruz) as described [72]. The immunoprecipitated DNA subjected to RT-PCR analysis with iTaq Universal SYBR Green Supermix (Bio-Rad) using the primer (SABiosciences, catalog no. GPM1030787(-)02A) that amplify a region of the mouse irf4 promoter containing the predicted FoxO1 binding sites. Data were analyzed with the LightCycler 480 software. The results were normalized to control IgG and input DNA.

### Adenoviral infection

Adenovirus construct that encoded FoxO1 mutants (FoxO1-TSS) is described elsewhere [73, 74] was a gift from Dr. T. Unterman (University of Illinois at Chicago). MH-S macrophages were infected with adenoviruses (1-50 multiplicity of infection [MOI]) and treated IL-4 after 24 h.

### Statistical analysis

Results are expressed as means ± SEM. Statistical analysis of significance was calculated by Student's *t*-test. Statistical significance is indicated in figure legends.
